# Wastewater treatment in tropical, vertical up-flow constructed wetlands with selected macrophytes species: A preliminary analysis

**DOI:** 10.1016/j.heliyon.2024.e41207

**Published:** 2024-12-12

**Authors:** Muhammedziyad Geleto, MihretDananto Ulsido, YohannesSeifu Berego

**Affiliations:** a Department of Water Supply and Environmental Engineering, Institute of Technology, Hawassa University, Ethiopia; b Department of Environmental Health, College of Medicine and Health Sciences, Hawassa University, Hawassa, Sidama Region, Ethiopia

## Abstract

The aim of this study was to investigate the growth characteristics of different local macrophyte species (n = 7) capable of growing in untreated coffee wastewater, select the dominant species for use in mesocosms, to study the efficacy of three major species in three replications (3 x 3) in improving the physicochemical characteristics of coffee wet mill wastewater, and to assess the contribution of macrophyte biomass to nutrient sequestration in the constructed wetlands. The current study showed that *Polygonumhydropiperoides(PH), Chrysopogonzizanioides (CZ), and Cyperusexaltatus(CE)* can sustain water logging and partially saturated conditions. The conducted wetland experiments pointed out the feasibility of VUFCW technology in ameliorating the impurities in wet coffee processing mills wastewater. Each of the three plant species has a peculiar ability to trap potential impurities. *Cyperusexaltatus*, for instance, is significantly efficient (α < 0.05) in reducing turbidity, minimizing PO_4_^3−^ and NO_3_^−^, and sequestrating it in the biomass as compared with the other two macrophytes. *Polygonumhydropiperoides* is good at improving DO; and *Chrysopogonzizanioides* is good at pH, EC, and TDS reduction. Therefore, the most effective constructed wetland can be designed by combining the three macrophytes vitalizing the ultimate potential of each of them in the system. However further studies and extended data collections are needed to ensure the enhanced quality of wastewater through the plant based treatment methods.

## Graphical abstract

**Figure:**
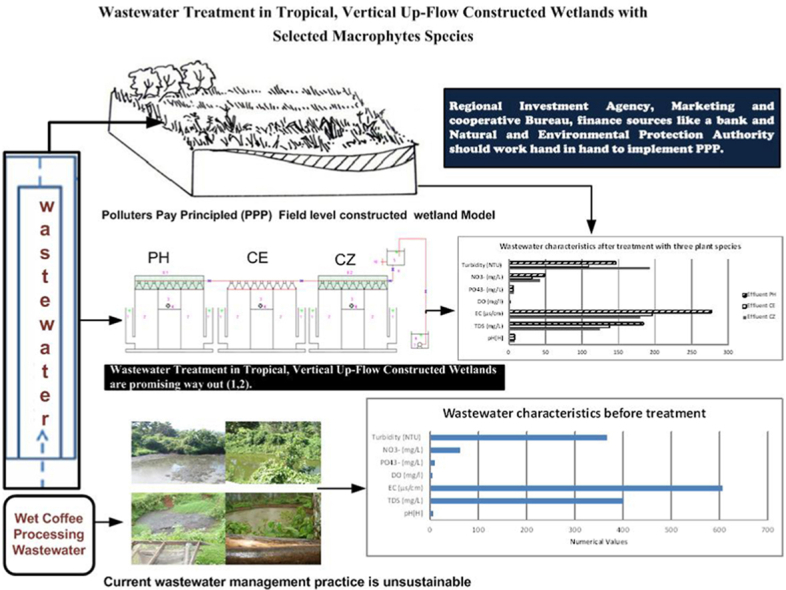

## 1 Introduction

The current study (2021/22) on Gidabo River water quality highlighted high levels of organics, turbidity, conductivity, nitrates, organic phosphates and low pH. According to a report from government sources, coffee producing wet mills are contaminating the water sources around the mill. The residents living around the wet mills are exposed to health risk due to pollutants released from the industries [1]. In small scale spatially distributed wet coffee processing industries of this watershed; the conventional wastewater treatment system may not be feasible due to shorter operational seasons, the limited capacity of wet coffee processing mills; operation and maintenance costs involved during the none operational season. In such situation, the application of constructed wetlands (CWs) offers an easy, cheap, and robust way-out for wastewater treatment in a country like Ethiopia where land is available at a reasonable price and the tropical temperatures are right for biodegradation [2]. These systems are capable of removing physical and chemical impurities in wastewater to a satisfactory level for reuse and discharge [3]. They have smaller operational and repair expenses [4]. Added extra benefits include “*tolerance against fluctuations of flow and pollution load, provision of habitat for many wetland organisms, and a more aesthetic appearance than conventional treatment structures*” [5,6].

Macrophytes play an imperative role in maintaining an aquatic ecosystem and act as bio-filters in constructed wetland technologies [1]. For instance, *Polygonumhydropiperoides* removes 74 % of nitrogen and 81 % of phosphates from a fish pond wastewater with high biomass productivity [2]; *Canna edulis*in warm seasonsremoves about 80 % of ammonium-nitrogen and 58–85 % total phosphates with elevated biomass productivity in treating combined urban and agricultural wastewater [3]; Vetiver grass (a *Chrysopogonzizanioides* family) and Saw-Sedge (a similar family with *Cyperusexaltatus*) removes about 65 % and 56 % total Kjeldahl nitrogen (TKN) from domestic wastewater [4] while cattails enhance sedimentation and flocculation in constructed wetlands [5]. Hence; rooted macrophytes in general serve as a living link between the substrate and the wastewater [6]. Besides playing an important role in an aquatic environment structuring, aquatic macrophytes are also known for their high biomass productivity [7,8] nutrient sequestration and removal capacity [2,4].

Vertical-flow constructed wetlands (VFCWs) with selected macrophyte species have shown promising results in wastewater treatment. Studies have highlighted the effectiveness of various macrophytes such as Paulownia, *Phragmitesaustralis*, Typha, and Phragmites in removing contaminants like COD, BOD_5_, nutrients, and pathogens from wastewater [[3], [4], [9], [10], [11]]. These plants not only enhance the treatment efficiency of VFCWs but also contribute to the biodiversity of the system, providing a sustainable and cost-effective solution for wastewater treatment in tropical regions [9]. Additionally, the physiological adaptation of ornamental tropical macrophytes like *Canna indica*, *Xanthosomarobustum*, and *Ruelliabrittoniana* to high concentrations of pollutants showcases their potential as phytoremediative plants in constructed wetland systems [12]. A review study showed that Typhalatifolia, Phragmitesaustralis, and Scripusvalidus as effective macrophytes for wastewater treatment in tropical vertical up-flow constructed wetlands, enhancing contaminant removal efficiency [13]. Studies done in India indicate that CWs can reduce biological oxygen demand (BOD), chemical oxygen demand (COD), and total suspended solids (TSS) by over 40 % [14]. The integration of various filter bed types enhances treatment outcomes; with specific combinations yielding better results for different contaminants [14]. The combined use of macrophytes and zooplankton in constructed wetlands has also demonstrated significant reductions in bacterial counts, BOD, and an increase in dissolved oxygen levels, further emphasizing the efficiency of this integrated approach in wastewater treatment [10]. CWs can also offer a sustainable and effective solution for treating coffee wastewater, leveraging natural processes to remove pollutants while providing ecological benefits [15,16]. Proper design, maintenance, and integration into the local environment are crucial for maximizing their performance and sustainability [17].

Albeit considerable experience exists worldwide, CWs technologies are still regarded as metaphorical black boxes. An empirical approaches has been used on CW design [18] using an effective surface area as a constraint or an easy first-order models [19,20]. However, Kadlec [18] documents those first-order decay models are insufficient for treatment wetlands design.

The intricate nature and significant volume of wastewater generated by coffee processing plant highlight the pressing need for efficient treatment solutions. In environments with limited resources, tackling pollutants at the mouth of a living water body, along with the requirement for affordable maintenance and operational cost of a treatment facility, becomes critical. Given these challenges, constructed wetlands present a viable option for wastewater treatment. So far very limited efforts have been made worldwide in general and particularly in Ethiopia for the application of wetlands for coffee processing wastewater treatment. Hence, the objectives of this research were to investigate the growth temperaments of different local macrophyte species capable of growing in untreated coffee wastewater, select the dominant species for use in mesocosms, to study the efficacy of three major species in improving the physicochemical characteristics of coffee wet mill wastewater, and to assess the contribution of macrophyte biomass to nutrient sequestration in the CWs.

## 2 Materials and methods

**Fig. 1:**
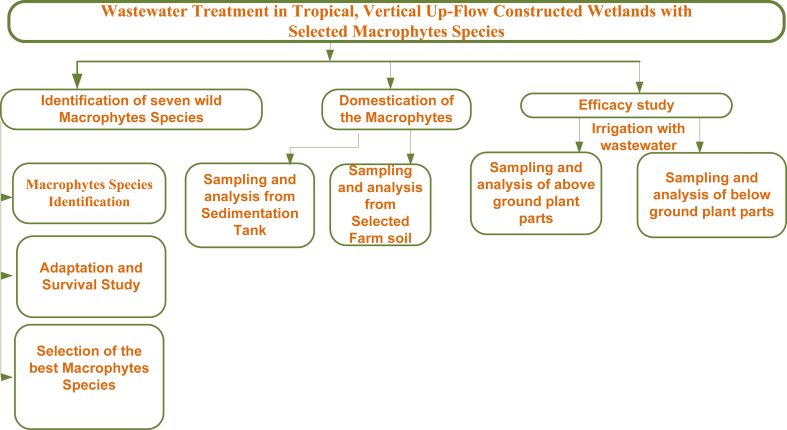
Schematics of the study.

Three steps were involved in a two years’ time:(a) to collect wild macrophytes adaptive to coffee wastewater, (b) domestication of the macrophytes under a controlled environment and (c) study the wastewater treatment efficacy of selected three macrophyte species under a natural environment (Fig. 1).

To investigate the growth temperaments of different local macrophyte species, 15–25 cm long sprouts of seven dominant macrophytes (Table 1) were collected in a week period from a natural population and prepared according toNayaket.al [21].

**Table 1:** Macrophytes names used for this study.

Macrophytesname	CODE	Common names	Local names	Source
*Glossostigmaelatinoides*	GE	Small Mud-mat	*Arem*	*Telamo-01 wet mill pond, Shebedino*
*Paspalidiumgerminatum*	PG	Egyptian Panic Grass	*Sare*	*Teremesa 102 wet mill pond, Shebedino*
*Bacopamonnieri*	BM	Water Hyssop	*Yemederabeba*	*Teremesa 102 wet mill pond, Shebedino*
*Polygonumhydropiper*	SW	Smart Weed	*Wefe'ankur or Lalute*	*Teremesa 102 wet mill pond, Shebedino*
*Polygonumhydropiperoides*	WP	Mild Water Pepper	*Aluma*	*Teshale wet mill pond, Lokabaya*
*Chrysopogonzizanioides*	CZ	Vetiver Grass	*Vetivar*	*WondoP.L.C wet mill pond, Aposto; Dale*
*Cyperusexaltatus*	CE	Spears of the Stream	*Qetema*	*Weneneta wet mill pond, Shebedino*

The collected sprouts of seven macrophytes were carefully washed with tap water and acclimated in clean water for three days as suggested by Ying et al. (2011) while changing the water every 24 h. The plants were selected for uniformity and were transferred to three 101.6 L polyethylene container (A = 0.442 m^2^, h = 0.3m) filled to 0.2 m depth with clean sand (D_50_ = 0.6 mm) having an effective porosity of 33 %. Each of the three containers holds 175 sprouts on an average area of 25cm^2^/plant, 25 from each macrophyte in three replications. All replications were irrigated with wastewater from *Kege* and *Weneneta* coffee wet a mill that is 47 km away from the experimentation site. The average concentration of NO_3_^−^-N and PO_4_^3-^-P of the wastewater were 74 and 3.83 mg/L respectively. Upon irrigation, the following six criteria namely [1]; the simplicity of cultivation [2], establishment rapidity [3], growth [4], tolerance of dry spells [5], nutrient storage capacity through biomass development, and [6] ecological acceptability were observed for comparison [21]. Each of the six criteria was given a score value ranging from 1 for *“poor”* to 7 for *“superior”* performing species [21] and aggregated as (Eq. (1)):

(1) $$TSV=\sum_{i=1}^{n}C_{i}$$

Where TSV is the total score value, n is seven, and C is criteria. After four weeks of phenotype observation, six plants were randomly selected for material analysis from each replication. The harvested macrophytes were carefully rinsed with tap water [21] and washed with deionized water, separated as living root and shoots, oven dried for constant weight in forced draft oven at 80 °C and then bulked for each species, with duplicate subsamples grounded and analyzed for biomass and nutrient. Those best-performing macrophytes at this level were selected for Vertical Up-Flow CW (VUFCW) efficacy study.

### 2.1 Wastewater treatment efficacy study

#### 2.1.1 Sample size, sampling time, and study period

**Fig. 2:**
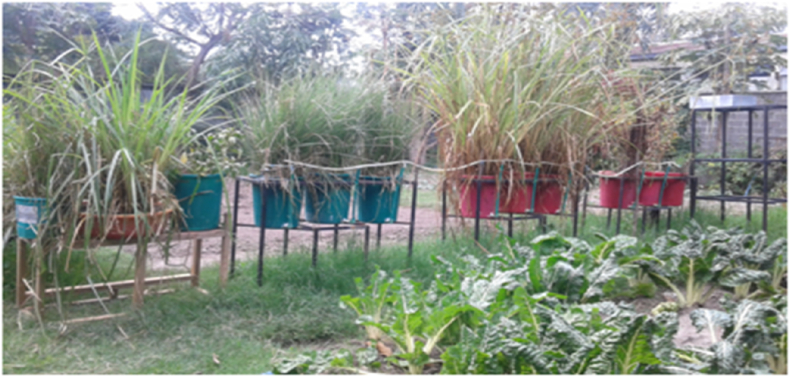
Actual setup of the mesocosm (three treatments and three replications).

Those three plant species selected after adaptation test were used for efficacy experiments. The mesocosm used for this study was an outdoor setup (Fig. 2) having a full access of the external environment. Three blocks each having three replications was constructed with big plastic pots (in triplicate blocks) to minimize errors. The three plants selected for this experiment were Mild Water Pepper (*Polygonumhydropiperoides*), Vetiver Grass (*Chrysopogonzizanioides*) and Spears of the Stream (*Cyperusexaltatus*). The plants were transferred to the mesocosms on the January 27, 2021 at four weeks of maturity. After 35 days of stabilization from November 1, 2021, the actual experiments on efficacy were conducted for about one month from December 21, 2021, to January 27, 2022.

#### 2.1.2 Schematics of the mesocosm

Each mesocosm was constructed (bottom to top) as follows (Table 2):

**Table 2:** Mesocosm replications, dimensions, materials and treatments used.

Mesocosms replication codes	Depth (bottom to top)		Treatments	Average plant density (number of stems/m^2^)
	Gravel^a^	Washed and clean sand^b^		
PT1-1	20 cm	50 cm,	*C. zizanioides*	16
PT1-2			*C. zizanioides*	
PT1-3			*C. zizanioides*	
PT2-1	20 cm	50 cm	*C. exaltatus*	16
PT2-2			*C. exaltatus*	
PT2-3			*C. exaltatus*	
PT3-1	20 cm	50 cm	*P. hydropiperoides*	16
PT3-2			*P. hydropiperoides*	
PT3-3			*P. hydropiperoides*	

a 10–20 mm diameter, effective porosity of 24 %.

b D_50_ = 0.6 mm, effective porosity of 33 %.

**Fig. 3:**
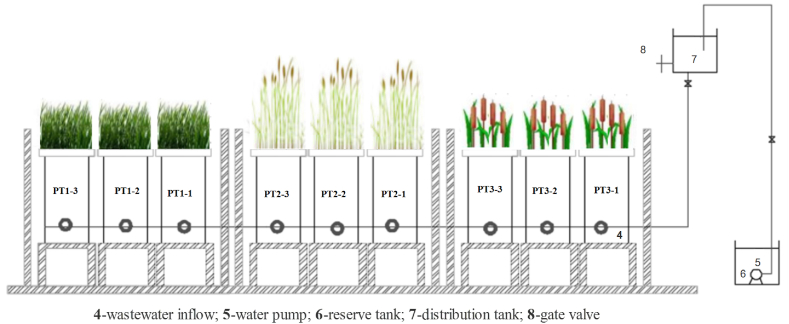
Schematics of the mesocosm.

The figure below (Fig. 3) shows the schematics of the experimental setup.

A barrel of 200 L capacity was erected within the premises of a forestry nursery site of Hawassa Agricultural College (7°3′ N, 38°28′E, 1674masl) where the mesocosm was installed. Wastewater from the barrel (influent) flooded each mesocosm with 0.55L of water per hour. This flow rate was arranged based on the physical property of the mesocosm. The wastewater was allowed to an average hydraulic retention time (HRT) of 48 h (Eq. (2)).

(2) HRT=v∗ε/Q

Where*V* is the volume of the mesocosm (79, 215.3 cm^3^),Q is the flow rate (550 cm^3^ h^−1^), ε is effective porosity of the mesocosm (0.33).

The hydraulic loading rate (HLR) was computed from (Eq. (3)) [22]:

(3) $$HLR=Q/A_{W}=550/1320.25=10cm/day$$

Where $A_{W}$ is the surface area of the mesocosm (1320.25 cm^2^).

Sampling from raw water (influent) and effluents from the mesocosm were done every 48 h of continuous irrigation. Wastewater samples from each mesocosm were collected in sterile 500 ml containers via individual effluent pipes. The containers were preserved in an ice box and transported from the field to Hawassa University Biosystems and Environmental Engineering School's Water Quality Laboratory located at a distance of 50m away from the field. Except the first week, samples were collated three times a week over one month period for water quality analysis.

#### 2.1.3 Water quality parameter

The water quality parameters used for profiling the influent and effluents were selected based on the intrinsic characteristic of coffee wastewater which includes dissolved oxygen (DO), organic phosphorous (PO4^−3^), Nitrate (NO_3_^−^), pH (hydrogen concentration), Total Dissolved Solids (TDS), Electric Conductivity (EC), and Turbidity. The methods, as well as the apparatuses used for the analysis in this study, were illustrated on Table 3 below.

**Table 3:** Investigated water quality parameters, methods, and apparatuses used.

Variables	Apparatuses
EC, pH, TDS	pH(pH meter, ELE international) & conductivity meter (SANXINSX713, Shanghai San-Xin Instrumentation Inc.)
PO_4_^−3^, NO_3_ˉ	Photometric measurements using Paqualab Photometer, ELE international
DO	DO analyzer JPS-J-605, Ningbo Biocotek Scientific Instrument Co., Limited
Turbidity	Turbidity meter, ELE international

The percentage reduction of the aqueous pollutants was calculated as (Eq. (4)):

(4) $$\text{Re}duction\left(\%\right)=\left(C_{i}-C_{e}/C_{i}\right)\times 100$$

where $C_{i}$ is the influent and $C_{e}$ is the effluent concentration of pollutants in mg.L^−1^ respectively.

### 2.2 Preparation and plant digestion

Plant samples were collected from nine sampling sites (Fig. 3) for plant biomass analysis. The plants were manually dug, washed properly with tap water, followed by distilled water to remove adsorbed soil particulates, trimmed carefully following the soil indicator line on the stock to separate root and shoot part of the plant, dry in a direct sunlight for more than 1 month first and finally an oven dry was done at 65 °C until constant weight is obtained. From the dry weight of the biomass of each plant tissue, a representative sample was pooled and ground to pass a 100-mesh sieve.

For plant tissue digestion, as described by Kassa et al. [23], 0.1 g plant tissue sample was pulverized using liquid nitrogen (100 mesh), 8 mL nitric acid was added to the sample and left overnight under Mars digestion (CEM recommended method), and in the next day 2 mL 30 % hydrogen peroxide (superior grade pure) was added to the plant samples.

### 2.3 Data analysis

Data analysis was done on IBM SPSS Statistics 20. Tukey-Kramer Honestly Significant Difference(HSD) test following single factor ANOVA F test was conducted at p < 0.05 level of significance.

## 3 Results and discussions

### 3.1 Biomass development

**Fig. 4:**
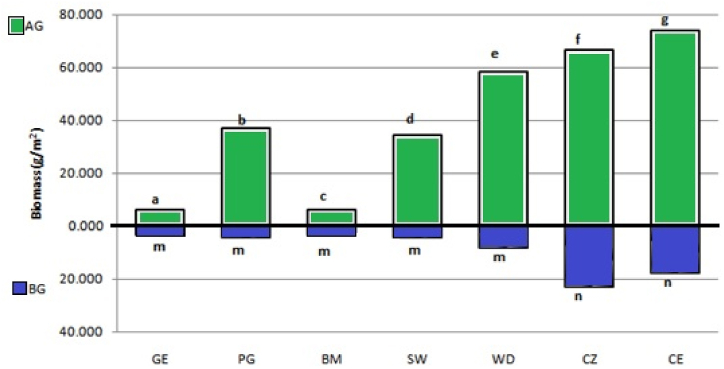
Mean below-ground (BG) and above-ground (AG) biomass (n = 21) of the seven species in g.m^−2^ after 30 days culture in coffee wet mills wastewater. Values with different subscripts are non-homogeneous at α < 0.05 Tukey-Kramer's HSD test.

All of the seven species showed different degrees of positive growth in the caffeine-rich coffee wastewaterfor a reason explained in a similarobservation made byNayak*et al.* [24]. They have not shown symptoms of toxicity and stunting. After 30 days of growth, mean species above ground biomass (Fig. 4) between 6 and 73.8 g/m^2^ and below ground biomass between 3.6 and 23.16 g/m^2^ (p < 0.001) were observed. *Glossostigmaelatinoides* (GE) and *Bacopamonnieri*(BM) showed low AG and BG biomass compared to the other species. Highest above-ground biomass levels (66.6–73.8 g/m^2^, p < 0.001) were reached by *Chrysopogonzizanioides* (CZ) and *Cyperusexaltatus*. *Cyperusexaltatus* showed a steady increase in shoot height and density over the trial period, as did *Chrysopogonzizanioides* and *Polygonumhydropiperoides*. The growth rates for the other species were gradually increased as the trial progress.

Maximum below-ground biomass was recorded for *Chrysopogonzizanioides*, 1.35 times higher than *Cyperusexaltatus,* comprised largely of the diffuse root system. Maximum above-ground biomass was recorded for *Cyperusexaltatus*1.11 times higher than *Chrysopogonzizanioides,* comprised largely of sharp leaf blades. Demars and Edwards, (2008) confirmed that macrophytes from nutrient-rich settings can hold higher proportion of nutrients in their biomass/tissue [25]. Many researches evidenced that biomass development capacity of macrophytes is directly correlated with the capacity of the macrophytes in removing nutrients from the wastewater [2,21,26]. Hence, from the current observation, it is possible to deduce that the total mean biomass production of 66.6,89.76 and 91.54 g m^−2^ (α < 0.05) realizedfrom*Polygonumhydropiperoides, Chrysopogonzizanioides and Cyperusexaltatus*respectively are an important indicator of their nutrient sequestration capacity in the reverse order. Additional agronomic aspects of each of the seven macrophytes were presented below.

#### 3.1.1 *Glossostigmaelatinoides*

The planting material is vegetative part of the plant and fragile to transport. It requires plenty of light to grow. In seven days, it establishes and starts to regenerate horizontally. The stands are very weak during their first few days. It is the second lower in biomass development (9.9 g m^−2^) and the least in below ground biomass production. Ecologically, it is a noninvasive weed species with no socio-economic benefits mentioned by the community. The plant can sustain complete water logging for a long time but weak in drought tolerance.

#### 3.1.2 *Paspalidiumgerminatum*

It is an aquatic, herbaceous, perennial grass that is propagated using either seed or sprouts. It is a useful wild pasture for livestock and wild aquatic animals. The grass is well adapted to quick re-growth on land re-flooded after a dry period. It is valued for growing first in wet coffee wastewater during the beginning of the rainy season. The grass is invasive and sometimes problematic in these environments. The macrophyte is ranked fourth in total biomass production of 41.4 g m^−2^ and similar with *Glossostigmaelatinoides, Bacopamonnieri, Polygonumhydropiper* and *Polygonumhydropiperoides* with its below ground biomass development. It sustains complete flooding of the root system for a long duration, but weak in drought tolerance.

#### 3.1.3 *Bacopamonnieri*

It is a slow-growing macrophyte that requires medium to high amounts of light. Since the plant took a longer time to establish and shade the ground, it is usually best planted in groups for better establishment. Propagation can be done either by seeds or stem fragments. The plant is resistant to drought and short flooded condition. If the light is not limiting, the plant will continue to grow horizontally until it reaches the nearby bunches. There is no known socio-economic benefit for the community.

#### 3.1.4 *Polygonumhydropiper L*

Locally called “*wefeankur*” in Amharic implies its preference by birds feeding on the white inflorescence. Albeit much consumption during the early stage of the plant leads bloating, animals also feed on this macrophyte. The plant can tolerate flooding but feeble in its tolerance towards dry season. There are two ways to propagate *Polygonumhydropiperoides* L.; either by seed or vegetatively by cutting.

#### 3.1.5 *Polygonumhydropiperoides*

A seasonal herb that reproduces mainly by seed, although broken stems can root at the nodes and grow into new plants. If the environment is favorable, it will establish well in less than two weeks. This plant tolerates short time flooding as well as prolonged dry season. There are no any socio-economic benefits known by the community.

#### 3.1.6 *Chrysopogonzizanioides*

Almost all vetiver species worldwide is vegetatively propagated. The planting material can be retrieved from underground stolons or from the vegetative part of the plant. Commonly used commercial genotypes of vetiver do not produce fertile seeds [27]. The plant is both aquatic and terrestrial. Once established, it sustains long dry and wet spells. The genotype used for the current study is a noninvasive species that can easily be controlled by the cultivation at the boundary of the hedge. Some of the ecological and socio-economic benefits cited by the community include its use for erosion control and forage for livestock during the early stage of the plant.

#### 3.1.7 *Cyperusexaltatus*

It is a macrophyte of bogs, water conveyance systems, and surface flowing water. Plant propagation can take place in both vegetative and seed form. The plant produces hefty light fine seeds capable of easily spreading along water courses. Hence, it is invasive and demands strict control. Ecologically, it is adaptive to a wide range of altitudes and can tolerate year-round water logged conditions. Its resistance to dry conditions is limited. During holidays and occasional celebrations, the plant is harvested and used for mating the ground surface.

Vetiver grass (*Chrysopogonzizanioides*), Spears of the Stream (*Cyperusexaltatus*) and Mild Water Pepper (*Polygonumhydropiperoides*) were better than the other species (Table 4) and found adaptable and better to be used to investigate their potential to filter-out impurities in wet coffee effluent wastewater.

**Table 4:** Summary of the species characteristic selected for VFCWLmesocosm experiment.

Species	Code	Criteria and Scores						TSV
		Cu.	Es.	To.	AG	BG	Ec.	
*Glossostigmaelatinoides*	GE	5	5	1	1	1	1	14
*Paspalidiumgerminatum*	PG	5	5	4	4	4	3	25
*Bacopamonnieri*	BM	1	3	4	2	2	2	14
*Polygonumhydropiper*	SW	5	3	4	3	3	5	23
*Polygonumhydropiperoides*	WP	5	3	6	5	5	5	29
*Chrysopogonzizanioides*	CZ	7	7	7	6	7	7	41
*Cyperusexaltatus*	CE	7	7	6	7	6	6	39

Cu.-Cultivation, Es.-Establishments, To.-Tolerance, AG/BG-Above ground/below ground biomass, Ec.-Ecology,Refer Table 1 for the codes listed on Column 2.

### 3.2 Performance characteristics of three major macrophytes

The investigation was conducted on the performance characteristics of the three major macrophyte species in removing nutrients and suspended solids (Table 5, Table 6). It was found that the plant species have shown different performances in remediating the impurities in the wastewater.

**Table 5:** Physicochemical properties of influent and effluent from VUFCWS after 61 days of maturity measured for four weeks.

Parameters	Influent	Effluent		
		*Chrysopogon zizanioide*	*Cyperus exaltatus*	*Polygonum hydropiperoides*
pH	6.67 ± 0.14	7.65 ± 0.17^a^^a^	7.91 ± 0.17^b^^a^	7.7 ± 0.16^a^^a^
TDS (mg/L)	401.47 ± 120.16	124.03 ± 12.05^a^	137.57 ± 10.72^b^	184.03 ± 12.3^c^
EC (μs/cm)	605.67 ± 86.12	179.78 ± 25.36^a^	196 ± 28.82^b^	277 ± 26.96^c^
DO (mg/l)	3.63 ± 0.62	0.68 ± 0.14^a^	1.32 ± 0.2^b^	0.36 ± 0.13^c^
PO_4_^3−^ (mg/L)	8.68 ± 0.08	6.27 ± 0.34^a^	5.81 ± 0.33^b^	6.33 ± 0.33^a^
NO_3_^−^ (mg/L)	61.37 ± 11.15	41.91 ± 4.95^a^	31.37 ± 5.19^b^	48.9 ± 4.95^c^
Turbidity	367 ± 71.06	193 ± 21.62^a^	108.99 ± 19.51^b^	146.01 ± 17.55^c^

Note: values were presented as average ± standard deviation, those with different superscripts are significantly different groups according to the Tukey test at α < 0.05.

a Represents values that increased.

**Table 6:** Recorded changes in physicochemical properties.

*Parameters*	*Quantity Reduction*			*% Reduction*		
	C.Z	C.E	P.H	C.Z	C.E	P.H
pH^a^	−0.98	−1.24	−1.03	−16.49	−11.69	−14.69
TDS (mg/L)^a^	277.47	263.86	217.47	69.11	65.72	54.17
EC (μs/cm)^a^	419.67	409.67	328.67	69.29	67.64	54.27
DO (mg/l)^a^	2.92	2.34	3.29	80.44	64.46	90.63
PO_4_^3−^ (mg/L)^a^	2.41	2.87	2.16	27.74	33.12	24.94
NO_3_^−^ (mg/L)^a^	19.46	30.00	12.47	31.72	48.88	20.32
Turbidity^a^	174.00	258.00	221.00	47.41	70.30	60.22

Note:C.Z- Chrysopogonzizanioides,C.E-Cyperusexaltatus, P.H.-Polygonumhydropiperoides, the minus sign indicates values that increased.

a Significant at α < 0.05.

The mean conductivity of the effluents ranged from 179.78 to 277 μS/cm. The lowest mean conductivity of 179.78 μS/cm was obtained for *Chrysopogonzizanioides* effluents followed by *Cyperusexaltatus* that was 196 μS/cm. The lowest mean TDS of 124.03 mg/L in *Chrysopogonzizanioides*effluent and relatively higher mean TDS in *Polygonum* hydropiperoides (184.03 mg/L), and *Cyperusexaltatus* (137.57 mg/L) effluents were recorded. The Turbidity in the effluent was lowest for *Cyperusexaltatus* (108.99 mg/L) and highest for *Chrysopogonzizanioides* (193 mg/L) with the highest variation (standard deviation = 21.62 mg/L).

The pH of the effluents varied from 7.65 to 7.91 where the highest mean pH of 7.91 was recorded for the effluent of the *Cyperusexaltatus* and the lowest mean pH of 7.65 was obtained for *Chrysopogonzizanioides*. The effluents dissolved oxygen concentrations varied from 0.36 to 1.32 mg/L. All effluents typically showed a critical anoxic environment created in the mesocosms.

The values of effluents PO_4_^3−^ were in the range of 5.81 ± 0.33to 6.33 ± 0.33 mg/L. The average concentration of PO_4_^3−^ was higher in the *Polygonumhydropiperoides* effluent (6.33 ± 0.33 mg/L) and lowest from *Cyperusexaltatus* effluents (5.81 ± 0.33 mg/L). The NO_3_^−^ concentration was higher in all effluents, in the range of 31.37 ± 5.19to48.9 ± 4.95 mg/L. The effluent from *Polygonumhydropiperoides*found to contain the highest concentration of NO_3_^−^. Relatively lower concentration of NO_3_^−^ was recorded in the effluent from *Cyperusexaltatus* with the highest variation. *Chrysopogonzizanioides* and *Polygonumhydropiperoides* are not significantly different(α < 0.05) regarding PO_4_^3−^removal and pH on their effluent levels.

Even though all the three wetlands recorded a decrease in turbidity, *Cyperusexaltatus* showed a higher rate of reduction as compared to the other two plant species due to its mat of sponge interwoven network of the fibrous root system that filtered out the upcoming suspended matters and utilized the dissolved matters in the wastewater. Both macrophytes significantly deteriorated the dissolved oxygen concentration as expected in this type of CW. Wetland plants are well known for their peculiar characteristics of taking dissolved oxygen through their aerenchyma tissue for respiration. Moreover, the oxygen changes are observed as a result of microorganisms activities and decay of dead plants on the soil surface [19]. That could be the reason for the observed significant reduction in the DO concentration of the effluents.

According to MDNR, (2016), *“conductivity is an indirect measure of the presence of inorganic dissolved solids such as chloride, nitrate, sulfate, phosphate, sodium, magnesium, calcium, iron and aluminum. The presence of these substances increases the conductivity of a body of water.”* [28]. Electric conductivity and total dissolved solid levels in all effluents sample have a reduction, ranging from 54.27 to 70.32 % and 54.16–69.11 % respectively. Both treatments are significantly different regarding EC and TDS reduction. The higher reduction regarding the turbidity level has impacted on the reduction of EC and TDS. Hence, these changes are influenced by the reduction of inorganic ions such as nitrate and phosphate present in the effluents [28]. The reduction of the concentration of dissolved solids may also be the cause of the reduction in dissolved oxygen concentrations.

Availability of nutrients for macrophytes in aquatic system is influenced by the pH of the water. pH is one of the limiting factor for aquatic life [28]. Too high or too low H^+^ or OH^−^ ions concentration affects biochemical reactions in a stream that threatens aquatic macrophytes [28]. Even though, its level is in the weak acid margin and influenced by a humic acid generated caused by the decomposition of organic matter, the pH level showed a marginal increase from its 5.67 ± 0.14 influent concentrations. The changes were influenced by atmospheric conditions, plants, and microorganisms activities. Generally, treatment wetlands maintain their effluents pH level between 6 and 7. There was a significant increase in all treatments. However, the highest percentage of incremental rate (16.49 %) and the lowest incremental rate (11.69 %) were observed in *Chrysopogonzizanioides* and *Cyperusexaltatus* effluents respectively.

The sample effluents from the three treatments showed a reduction in nitrate-nitrogen ranging from 20.32 % in *Polygonumhydropiperoides* to 48.88 % in *Cyperusexaltatus*. *Polygonumhydropiperoides* showed relatively smaller reduction as compared with the other two macrophytes and lower efficiency of the laboratory experiment done using the same macrophyte reported by previous study [2]. The reason could be the difference on the set-up of the experiment, the HLR and the nature of the coffee wastewater as compared with fish pond wastewater used in their case. Nitrate (NO_3_^−^) reduced in *Chrysopogonzizanioides* effluents by 19.46 mg/L (31.72 %), *Cyperusexaltatus* effluents by 30 mg/L (31.72 %) and *Polygonumhydropiperoides* effluents by 12.47 mg/L (20.32 %) which is better as compared with most researchers conducted on vertical flow CWs [2,4,29] taking into account the HLR and the experimental setup. Microbiologically mediated anaerobic conditions in the wetland are responsible for the reduction of nitrate by converting it into nitrogen gas [30]. The process was described by [31]; and [30] in the redox-reaction (Eq. (5)) of a denitrification process as a result of the anoxic conditions created which is evidenced in the current research by the reduction of the concentration of H^+^ ion (hence increased pH).

(5) $$5{\text{CH}}_{2}O+N{O_{3}}^{-}+4H^{+}\;\to \;5{\text{CO}}_{2}+7H_{2}O+2N_{2}$$

When nitrate concentrations become excessive, along with another essential nutrient like phosphorus exists in excess concentration; biomass production and respiration can also be the reason for the reduction that is evidenced through the depleted DO in the system.

Root biomass differences among the macrophytes played the role on the variation between the macrophytes on their nutrients sequestration capacity. The well-established root system of *Chrysopogonzizanioides*and*Cyperusexaltatus*) facilitated the NO_3_^−^ removal by offering carbon from exudates and decomposition to denitrifiers [[32], [33], [34]]. The interwoven blankets of roots developed by *Chrysopogonzizanioides*and*Cyperusexaltatus* provided an oxygenated zone where NO_3_^−^ can distribute out of the root zone to the adjoining anoxic layer where denitrification can occur [35].

Similar to Nitrate (NO_3_^−^), phosphorus is a limiting factor for plant development [29]. Phosphorus usually occurs in nature as phosphate (PO_4_^−3^). PO_4_^−3^ decreased in *Chrysopogonzizanioides* effluents by 2.41 mg/L (27.74 %), in *Cyperusexaltatus* effluents by 2.88 mg/L (33.12 %), and in *Polygonumhydropiperoides* effluents by 2.16 mg/L (24.94 %). Albeit the efficiency look low as compared with most reports [2,4,29], the mass abstraction rate is relatively high. This was as a result of phosphates sorption on the fine sand sediment and uptake by the macrophytes. The improved pH conditions facilitated the process as phosphate solubility is decreased through alkalinity and the limited air entry into the lower reaches of the system induced anaerobic conditions that immobilized the nutrient [36,37].

### 3.3 Nutrients in the biomass

**Fig. 5:**
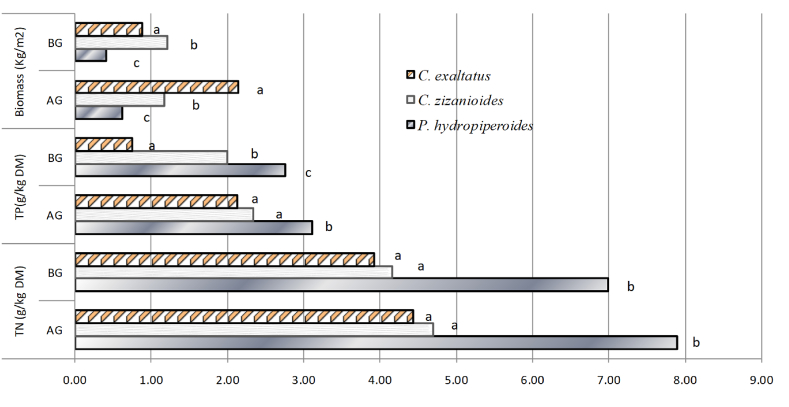
Mean below-ground (BG) and above ground (AG) biomass in kg.m^−2^, total phosphorous and total nitrogen in g.kg^−1^ of DM (n = 9) of the three macrophytes after 90 days of cultivation irrigated with coffee wet mills wastewater. Values with different subscripts are non-homogeneous at α < 0.05 Tukey-Kramer's HSD test.

*Cyperusexaltatus* and *Chrysopogonzizanioides-*based mesocosms showed superior above ground(AG) and below-ground(BG) biomass yield (α < 0.05) respectively (Fig. 3). However, the statistical analysis did not identify significant differences among the AG and BG biomass of *Cyperusexaltatus*(α = 0.996). Significantly higher total nitrogen in g.kg^−1^ of DM in AG biomass of *Polygonumhydropiperoides* in one kg of dry matter (DM) was detected compared to the other two (α < 0.01). However, the concentrations of N in the AG stocks of the *Cyperusexaltatus* and *Chrysopogonzizanioides* were not statistically significant (α = 0.762) (Fig. 5).

The concentrations of P found in the AG parts of *Polygonumhydropiperoides* (Fig. 3) were significantly higher (α < 0.01). Furthermore, the statistical analysis showed that the accumulation of P in the BG parts of all the three plant varied significantly (α < 0.05). On the other hand, the concentrations of P in the AG biomass of *Cyperusexaltatus* and *Chrysopogonzizanioides* were not statistically significant (α = 0.151). Significantly higher N and P concentrations (α = 0.01) were sequestered in *Polygonumhydropiperoides* (Fig. 3). The amount of P and N in the AG biomass found in this study were *Cyperusexaltatus*(2.15 g/m^2^, 6.29 g/m^2^), *Chrysopogonzizanioides*(2.58 g/m^2^,5.27 g/m^2^) and *Polygonumhydropiperoides*(1.5 g/m^2^, 3.82 g/m^2^) respectively, shows that due to their higher biomass productivity, the former two macrophytes sequestrated more N and P per unit area as compared with *Polygonumhydropiperoides*. The three macrophytes were developed relatively longer and dense root systems when allowed to grow on polluted wastewater supplied below the root system as compared with the one reported by Refs. [2,4,26]. Compared with reported literature P and N sequestration values for common-reed [36,37] and reed-canary-grass [38] cultivated in horizontal flow wetlands, the three macrophytes in the current VUFCWs showed better nutrient removal and accumulation performances. Similar to the observations reported by the previous study [21,38] it is noted that non-significant differences was observed among the macrophytes in phosphorus absorption performances per unit area that varies only between 1.5 and 2.58 g/m^2^, but they significantly differed in their capacities to accumulate nitrogen between 3.82 and 6.29 implicated that nitrogen is a limiting nutrient that affected the yield of macrophytes in CWs and control phosphorus accumulation by wetland plants.

## 5 Conclusions

The current adaptation study showed that water logging is not affected the growth performance of the three species. The plant tolerated the toxic effluents applied at different sequences. Vegetation in the wetland provides a substrate (roots, stems, and leaves) upon which microorganisms can grow as they break down organic materials. The plants improves the dissolved oxygen concentration which is a crucial parameter for living organisms in the aquatic environment by 80.44 % (*Chrysopogonzizanioides)*, 90.63 % (*Polygonumhydropiperoides)*and64.46 % (*Cyperusexaltatus*) for the same ecological condition. Particularly *Cyperusexaltatus*is superior in turbidity reduction (70.30 %). It is recommended to use the three species in combination to optimize pollutant sequestration. Moreover, further research is recommended to optimize the system and see the plants performance on the field. Underground seepage, soil retention, and dilution effect of rainfall is overlooked in the current nutrient balance study. Moreover, climate and seasonal variability were undermined. Since pollutant concentrations can vary widely in the tropics, such variability was not considered in the current study. Future studies shall tackle this limitation by conducting the experiment in a growth chamber. For future work, long-term monitoring at least for three consecutive seasons is suggested to see the level of sequestration saturation limits of the wetlands.

The current research has led to the following policy recommendationson wet coffee processing industriesand areas in the country [1]: Combined use *of Chrysopogonzizanioides, Polygonumhydropiperoides,* and*Cyperusexaltatus.* [2] Implement an Integrated Water Resource Management (IWRM) approach that shall incorporate constructed wetlands as viable solutions for wastewater treatment and ecosystem restoration [3]. Capacity building, resources, and training for local communities, engineers, and technicians to build capacity for custom designing, implementing, and maintaining constructed wetlands effectively [4]. Encourage research, development, and collaboration between academic institutions, government agencies, and NGOs to conduct localized research on constructed wetlands, particularly focusing on plant and microbial interactions and pollutant dynamics [5]. Introduce funding mechanisms, tax incentives, or subsidies to support the establishment and maintenance of constructed wetlands, encouraging community stewardship and ownership [6]. Establish clear guidelines and policies to support the integration of constructed wetlands into existing wastewater management frameworks and ensure compliance with environmental standards [7]. Foster interdisciplinary collaborations that encompass ecology, engineering, social science, and policy to ensure holistic approaches to constructed wetland research and application. In summary, while constructed wetland studies in the tropics can encounter significant challenges, thoughtful policy measures can enhance their feasibility and effectiveness, leading to sustainable water management solutions.

## CRediT authorship contribution statement

**Muhammedziyad Geleto:** Writing – review & editing, Writing – original draft, Supervision, Software, Methodology, Formal analysis, Data curation, Conceptualization. **MihretDananto Ulsido:** Supervision, Resources, Project administration, Methodology, Investigation, Funding acquisition, Formal analysis, Data curation, Conceptualization. **YohannesSeifu Berego:** Writing – review & editing, Writing – original draft, Visualization, Validation, Supervision, Software, Resources, Methodology, Investigation, Formal analysis, Data curation, Conceptualization.

## Data availability statement

Data included in article/supp. material/referenced in article

## Declaration of competing interest

The authors declare that they have no known competing financial interests or personal relationships that could have appeared to influence the work reported in this paper.
